# Grey Relational Analysis and Grey Prediction Model (1, 6) Approach for Analyzing the Electrode Distance and Mechanical Properties of Tandem MIG Welding Distortion

**DOI:** 10.3390/ma16041390

**Published:** 2023-02-07

**Authors:** Hsing-Chung Chen, Andika Wisnujati, Mudjijana, Agung Mulyo Widodo, Chi-Wen Lung

**Affiliations:** 1 Department of Computer Science and Information Engineering, Asia University, Taichung 413, Taiwan; 2 Department of Medical Research, China Medical University, Taichung 404, Taiwan; 3 Department of Automotive Engineering Technology, Universitas Muhammadiyah Yogyakarta, Yogyakarta 55183, Indonesia; 4 Department of Mechanical Engineering, Universitas Muhammadiyah Yogyakarta, Yogyakarta 55183, Indonesia; 5 Department of Computer Science, Universitas Esa Unggul, Jakarta 11510, Indonesia; 6 Rehabilitation Engineering Lab, University of Illinois at Urbana-Champaign, Champaign, IL 61820, USA; 7 Department of Creative Product Design, Asia University, Taichung 413305, Taiwan

**Keywords:** aluminum AA5052, tandem MIG welding, grey relational analysis, grey prediction model GM (1, 6)

## Abstract

The tandem metal inert gas (MIG) process uses two wires that are continuously fed through a special welding torch and disbursed to form a single molten pool. Within the contact tip of the modern approach, the wires are electrically insulated from one another. This study identified the effect of welding electrode spacing on the distortion of AA5052 aluminum plates and different mechanical properties including hardness and thermal cycle using grey relational analysis. Plate distortion was subsequently predicted using the grey prediction model GM (1, 6). This research used a pair of 400 mm × 75 mm × 5 mm of AA5052 plates and electrode distances of 18, 27, and 36 mm. The welding current, voltage, welding speed, and argon flow rate were 130 A, 23 V, 7 mm/s, and 17 L/min, respectively. The temperature was measured using a type-K thermocouple at 10, 20, 30, and 40 mm from the center of the weld bead. The smallest distortion at an electrode distance of 27 mm was 1.4 mm. At an electrode distance of 27 mm, the plate may reach a proper peak temperature where the amount of heat input and dissipation rate are similar to those for electrode distances of 18 mm and 36 mm. The highest relative VHN of 57 was found in the BM, while the lowest, 46, was found in the WM, showing good agreement with their respective grain sizes. Six parameters were designed using grey relational analysis (GRA) and subsequently employed in the grey prediction model GM (1, 6). Process evaluation results show that predictions for welding distortions are consistent with actual results, thus, the GM (1, 6) model can be used as a predictive model for welding distortions of 5052 aluminum plates.

## 1. Introduction

Welding is a metal joining method that is widely employed for aluminum and its alloys. Metal inert gas (MIG) welding is a welding process that is generally used to connect aluminum plates [1]. MIG welding uses noble gas (inert gas) to protect the electric arc while the electrode wire is fed continuously by an electric motor so that the welding process can be done semi-automatically or automatically. MIG welding creates the welding arc by continuously regulating metal from the consumable wire electrode. In terms of weld-bead shape and mechanical reliability, welding process factors are also very important in defining weld joint quality [2,3]. Furthermore, Adin discovered that changes in the current and voltage values of MIG welded joints on carbon steel have a significant effect on the tensile strength and elongation values of the joints, increasing the toughness of the MIG welded material [4]. In addition to fabrication, the benefits of the welding process can also be utilized for repairs, such as filling holes in castings, creating a welded layer on tools, thickening worn parts, and a variety of other repairs. The imperative for efficient manufacturing construction has resulted in a new trend in manufacturing design characterized by the use of thin plates. However, the use of welding processes such as MIG for joining thin plates frequently results in severe weld distortion due to local expansion and contraction of the weld metal and its adjoining area during welding [5]. When aluminum alloys are used for welded structures, the situation worsens due to their undesirable thermal material properties, such as a high thermal expansion coefficient, which causes significant distortion.

As a result, welding distortion can result in out-of-tolerance geometry, lowering product quality and having an adverse effect on the buckling strength of welded structures [6,7,8]. Various MIG welding methods have been developed to improve welding results, one of which is tandem welding. For several decades, the scope of use of tandem MIG welding techniques in construction has been very wide, because it can reduce process time in manufacturing industries, including shipping, bridges, steel frames, pressure vessels, rapid pipes, pipelines, and so on [9,10,11]. Tandem MIG welding is the best method to increase welding productivity. Tandem MIG welding is considered to have high production effectiveness that can be achieved with high welding speeds [12]. It consists of two independent electrodes (early electrode and delayed electrode) positioned parallel to the weld line and controlled individually under distinct welding conditions (current and voltage) [13]. The main advantages of the tandem MIG welding method are its extremely high efficiency, low heat input, improved weld seam integrity, and extremely high deposition rate. However, it can give rise to distortion known as longitudinal or buckling distortion due to the occurrence of the expansion process during the welding process and shrinkage when the weld begins to cool [14]. Over the last few decades, studies have developed several methods of planning and controlling this distortion [15,16]. In general, control of welding distortion and residual stresses can be accomplished using mechanical effects, thermal effects, or a combination of the two techniques and can be performed either during welding or after [17,18,19].

Local industry, however, is struggling with a lack of recognition and oversight of the input process factors required to create a quality weld joint that matches the required specifications [20,21]. Traditional processes for determining welding parameter settings currently comprise an empirical method of trial and error, which is a time-consuming and error-based design process. Wu suggested that mechanical and thermal balance techniques be developed during the welding process to control welding distortion by reducing welding thermal gradients and producing a tensile stress field near the welded region. A review of these approaches would be beneficial in order to fully understand the main mechanism of mitigating welding-induced distortion and to select the most appropriate method for minimizing deformation based on realistic fabrication demands [22]. However, welding distortion can be precisely predicted during the manufacturing process while accounting for both local shrinkage and root gap. Mitigating weld distortion to meet product requirements is a difficult task because welding shrinkage cannot be avoided, only controlled. An increase in electrode distance is associated with a decrease in temperature at the center of the welding arc. The determination of the distancing of welding electrodes in tandem MIG welding depends on the type of electrode, the diameter of the electrode core, the material being welded, the geometry of the connection, and the accuracy of the connection [23,24].

Thus, this study proposed using a GRA and GM (1, 6) approach and determined its validity not solely for the analysis of empirical data for MIG welding but also for predicting the distortion of tandem MIG welding in aluminum AA5052 plate. Thus, this research contributes to:

- Determining the influence of mechanical properties parameters and the variable of electrode distance using GRA analysis on the distortion of aluminum AA5052.
- Derive differential equations from empirical data of experimental results to predict plate distortion values of AA5052 using GM (1, 6) based on the parameters resulting from the GRA analysis.
- The value obtained from solving the complete differential equation of the GM (1, 6) prediction model has a value that is broadly similar to the results of the empirical distortion test, so it is useful for the industrial world or users to optimize the parameters that affect the distortion of welding results.

In terms of its relationship to the voltage from the welding current, it can be said that the distance between the welding electrodes and the welding speed are largely independent of the welding voltage but are directly proportional to the welding current so that the effect of the electrode distance on the back bead occurs if the distance is short. In this case, the welding bead will coalesce. The challenge with welded joints is that their mechanical characteristics are inferior to those of the base material. Mechanical properties using microhardness-Vickers, distortion, and thermal cycles in welding temperatures are the output responses evaluated in the study to analyze using the GRA and GM (1, 6) approach based on the effect of variable electrode distance on tandem MIG welding.

## 2. Related Works

Welding is a manufacturing process in which two or more similar or dissimilar materials are permanently joined by coalescence formation with or without the application of external pressure, heat, or filler material. Weld-bead formation does not require the fusion of the faying surfaces of the base materials [25]. Welding processes are broadly classified into two groups based on whether or not the base material fuses: solid-state welding and fusion welding. Fusion welding occurs when the faying surfaces of the base materials and filler material melt together to form a weld bead. Conversely, solid-state welding occurs when no such melting occurs during the welding process. The following types of welding techniques are available in the manufacturing industry. Each of these welding techniques has its advantages as shown in Table 1.

The material aluminum AA5052 has a process of reduction of 87 percent, resulting in a tensile strength of 325 MPa and a strain of 2.5%. After annealing treatment at 300 °C for 4 h, the elongation is strengthened to 23%, but the tensile strength is reduced to 212 MPa [26,27]. Aluminum AA5052 is an alloy that is not heat treated (non-heat treatable); thus, the strengthening mechanism is carried out by means of a solid solution and cold working. Similarly, aluminum from the AA7075 (a magnesium-zinc alloy) and 6000 series, and AA6082 (a magnesium-silicon alloy), are also commonly utilized in aviation applications due to their excessive specialized strength, hardness, and resistance to corrosion at cleft temperatures [28,29,30].

Prakash et al. designed the multi-response optimization problem solved by using the GRA method [31]. This is the most often utilized optimization strategy. The parameters are processed, the outputs are observed, and the results are tabulated. This system provides data conditions and facilitates decision-making. Adin claimed that optimizing the welding parameters used in friction welding to join AISI steel bars is quite important. Furthermore, the effects of welding parameters on tensile strength and axial shortening were investigated, and welding parameters were optimized using the Taguchi method to achieve high-quality weld joints [4,32]. The GRA approach is used to solve complex problems. In this paper, we will obtain a GRG through the GRA process, which is used for problem evaluation. The GRA approach relates to the calculation of all the effects of multiple aspects and their correlation and is also called the straightening of factor relations. The GRA approach utilizes relevant data from the grey system to quantitatively compare each element according to the level of resemblance and variation between factors in order to determine their relationships [33,34].

Qazi et al. integrated a methodology of GRA combined with primary component analysis (PCA) for the optimization of the shielded metal arc welding (SMAW) technique of steel plates SA 516 grade 70. The authors studied the effect of SMAW specifications on the transformation of mechanical characteristics [35]. They reported that the experiments display acceptable agreement with optimum outcomes. According to Cai et al. [36], the welding position greatly affects the weld pool due to the effect of gravity, where, in a flat position, the surface of the pool becomes concave, while in a vertical position, the pool surface is even more concave due to more flowing filler metal on the welding pool. Previous studies used MS plate (Grade: IS 2062) specimens to solve multi-objective optimization issues in the metal inert gas (MIG) welding process. The specimen was investigated to find the best combination of input elements such as welding current, open circuit voltage, and plate thickness to achieve superior weld strength and bead geometry quality criteria such as tensile strength, bead width, reinforcing, and penetration. Sahu et al. used GRA and PCA to look at how multiple objectives turned into specific feedback. The goal was to find the best way to set the relationship between the input factors [37]. GRA is a method for calculating the degree of correlation within sequences and combining several output responses into a single output response by applying a GRG to each output response [38,39]. Researchers [40,41,42,43,44] used GRA to optimize grey-based Taguchi analysis (GTA) welding process variables for the multi-objective response by GRG. Taguchi’s GRA multi-objective optimization approach was used in this study to optimize gas tungsten arc welding (GTAW) characteristics of various AA6061 and AA2014 alloys. Chavda et al. [45] studied the MIG welding process as well as Taguchi’s DOE Method. They concluded that the main factors in the GMAW process are current, voltage, speed, inert gas, gas flow rate, wire feed rate, wire diameter, and so on, and that these variables will affect the various weld properties. Kulkarni et al. [46] investigated the welding parameters of MIG welding by GRA employing an L32 orthogonal array. Several performance factors, including torch angle, wire feed rate, standoff distance, welding speed, and welding current, were optimized using a Taguchi L32 array and grey relational analysis. GRA is used for optimization because it is the most effective strategy for multi-response optimization. Material removal rate (MRR) and surface finish are influenced the most by feed rate, according to the literature [47,48].

## 3. The Proposed Method and Materials

### 3.1. Data Acquisition

**Figure 1:**
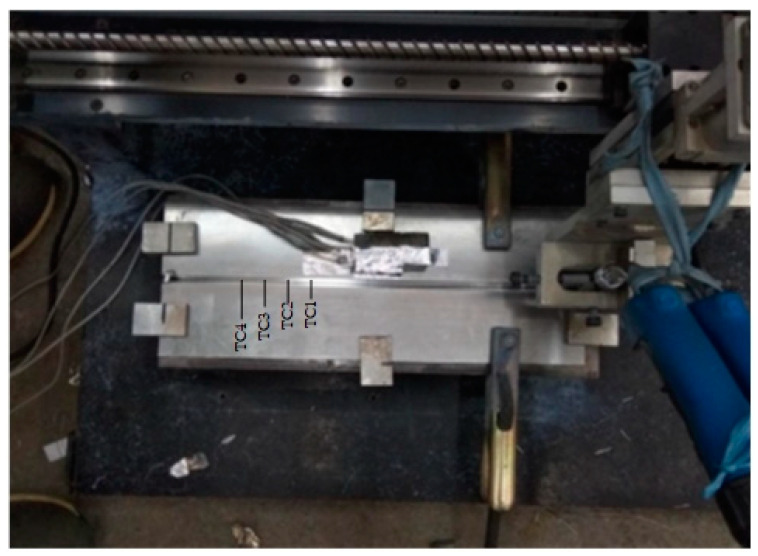
Semi-automatic welding tool.

**Figure 2:**
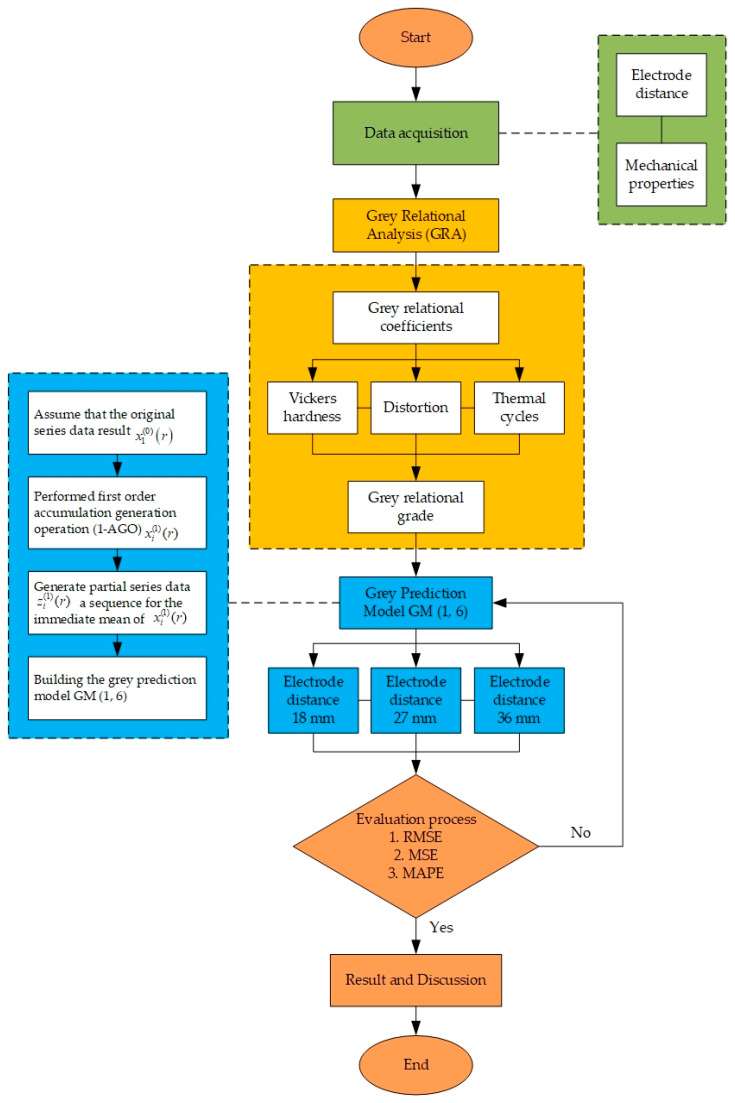
System architecture.

The input data for this method are the welding electrode distance and mechanical properties of a MIG welding experiment. For conducting the experiments, electrode distances of 18, 27, and 36 mm, and a welding speed of 7 mm/s, are chosen as input parameters. The welding speed was selected by trial and error using dummy specimens, whereas the electrode distances were based on Mudjijana et al. [49,50]. The mechanical properties used in this study are Vickers hardness, distortion, and thermal cycle, which are used to determine the distortion effect of the AA5052 aluminum plate. Figure 1 depicts a semi-automatic welding tool that can be used to place specimens and adjust the welding speed, the distance between the electrode and the specimen, the welding gun position, and the welding gun movement. Our method is illustrated in Figure 2, which shows the whole system architecture.

### 3.2. Materials

In this study, a pair of aluminum AA5052 materials 400 mm × 75 mm × 5 mm in size, with a V (70°) grove, and 2 mm root were welded using MIG tandem welding, and 0.8 mm diameter ER5356 electrodes. Aluminum AA5052 series aluminum alloys provide good weight-to-strength ratios, corrosion resistance, weldability, and recycling possibility. This alloy has excellent workability, excellent corrosion resistance, excellent weldability, and moderate strength. As a result, it is used in aviation fuel or oil lines, gasoline tanks, various modes of transportation, sheet metal work, appliances and lighting, wire, and rivets [51]. Welding was done with a Tenjima 200S MIG welding machine with leading and trailing torches tilted at an angle of 80°, a current of 130 A, a voltage of 23 V, a feeding rate of 25 mm/s, an arc length of 10 mm, and a flow rate of 17 L/min of argon gas. The tandem MIG welding process does not use external cooling. This is because the increase in cooling causes a change in the weld fusion line [52].

**Figure 3:**
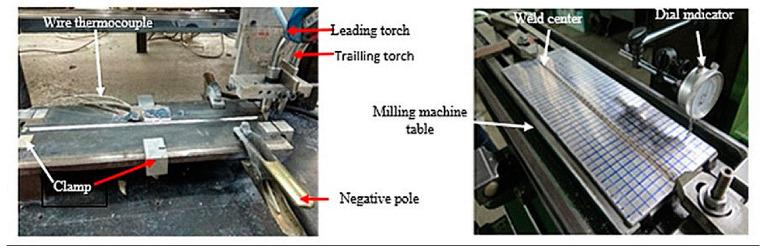
Setup for tandem MIG welding AA5052.

The output of this MIG welding is measurements of the distortion of the welding joints on the aluminum AA5052 plate. As illustrated in Figure 3, distortion was evaluated after welding was completed using a dial indicator with 0.01 mm accuracy. The welding temperatures of the thermal cycle (TC), TC1, TC2, TC3, and TC4, were measured at 10, 20, 30, and 40 mm from the center of the weld bead using a type-K wire thermocouple, an ADAM-4561 data acquisition module, and verified using a computer.

### 3.3. Grey Relational Analysis Procedure

GRA can handle multi-response optimization problems in the presence of incomplete and unclear information. GRA is used to obtain the value of GRG to evaluate multiple responses, allowing the optimization of complex multiple responses to be transformed into the optimization of a single response with the GRG [36]. The welding process includes several responses, and welding quality is heavily dependent on maximizing all of these at the same time. As a result, researchers typically use GRA to analyze diverse responses at the same time [18,37]. GRA is part of the grey system theory and aims to analyze the grey relational degree between each factor in the grey system [53].

The detailed steps of the GRA method are as follows:

Step 1: Standardized data transformation.

GRA functions as a discovery concept, assembling known and unknown components to achieve the best level of reaction. Grey relational coefficients (GRC) and GRG are calculated with the help of value normalization.

- Benefit-type factor

A factor with a greater value than the original data yields better quality characteristics (larger-the-better) as shown in Equation (1).

(1) $$x_{i}^{*}(k)=\frac{x_{i}^{\left(0\right)}(k)-\min x_{i}^{\left(0\right)}(k)}{\max x_{i}^{\left(0\right)}(k)-\min x_{i}^{\left(0\right)}(k)}$$

- Defect-type factor

A factor with a lower value than the original data yields higher-quality characteristics (smaller-the-better) as shown in Equation (2).

(2) $$x_{i}^{*}(k)=\frac{\max x_{i}^{\left(0\right)}(k)-x_{i}^{\left(0\right)}(k)}{\max x_{i}^{\left(0\right)}(k)-\min x_{i}^{\left(0\right)}(k)}$$

- Medium-type or nominal-the best

Factors that have the same value or are closest to the specified value standard show better quality characteristics, as shown in Equation (3).

(3) $$x_{i}^{*}(k)=1-\frac{\left|x_{i}^{\left(0\right)}(k)-x_{tv}^{\left(0\right)}\right|}{\max\left(\left(\max x_{i}^{\left(0\right)}(k)-x_{tv}^{\left(0\right)}\right),\left(x_{tv}^{\left(0\right)}-\min x_{i}^{\left(0\right)}(k)\right)\right)}$$

where $x_{i}^{*}(k)$ is the data after grey relational generation, $\max x_{i}^{\left(0\right)}(k)$ is the maximum value of the original sequence factor, $\min x_{i}^{\left(0\right)}(k)$ is the minimum value of the original sequence factor, and $x_{tv}^{\left(0\right)}$ is the target value.

Data standardization is a stage in grey relational generation, where the experimental results are normalized to a value on a scale of 0 to 1 due to different units of measurement. Data pre-processing converts original sequences into a set of comparable sequences [53].

Step 2: Calculating the deviation sequence.

Using the following formula, the deviation sequence calculation in Equation (4) attempts to estimate the actual difference between the compared series and the reference series.

(4) $$\Delta_{0i}(k)=\left|x_{0}^{*}(k)-x_{i}^{*}(k)\right|$$

where $\Delta_{0i}(k)$ is the deviation sequence, $x_{0}^{*}(k)$ is the reference sequence, and $x_{i}^{*}(k)$ is the comparability sequence.

Step 3: Calculating the grey relational coefficient

The calculation of the GRC in Equation (5) is carried out to determine the sequence with the lowest deviation using a discriminating coefficient between 0 and 1. In general, the discriminating coefficient is 0.5. The sequence with the lowest deviation will produce the GRC with the highest value, which is “1”.

(5) $$\xi_{i}(k)=\frac{\Delta_{\min}+\xi \Delta_{\max}}{\Delta_{0i}(k)+\xi \Delta_{\max}}$$

where $\xi_{i}(k)$ is the grey relational coefficient, ξ is the distinguishing coefficient (0.5), $\Delta_{\min}$ is the minimum deviation sequence, and $\Delta_{\max}$ is the maximum deviation sequence.

Step 4: Calculating the relative grey relational grade

GRG represents the degree of correlation between the reference and comparative sequences in Equation (6). The higher the value of GRG, the stronger the correlation between the reference and comparability sequences. GRG calculation is done using the following formula.

(6) $$r_{i}=\sum_{i=1}^{n}\left(w\left(k\right)\times \xi_{i}(k)\right)$$

where $r_{i}$ is the grey relational grade and $w\left(k\right)$ is the quantity *of the number k influence factor.*

### 3.4. Existing Grey Prediction Model GM (1, N)

The accuracy of the current grey prediction model GM (1, N) cannot be predicted because of the solution’s inaccuracy. It is improper to solve the existing GM (1, N) with the assumption that the related series first-order accumulated generating operation data are constants. GM type (1, N) is an extension of GM (1, 1). In this study, we proposed a new grey prediction model GM (1, 6). This model is extremely useful when historical data is unavailable. However, the model requires at least four periods of time of historical data.

The steps to generating the GM (1, 6) model are described below.

Step 1: Assume that the original series of data came from determining how welding distortion changed over time as a series of dependent variables.

Build the original data series in chronological order in Equation (7) for the dependent variables and Equation (8) for the independent variables.

(7) $$x_{1}^{\left(0\right)}\left(r\right)=\left\{x_{1}^{\left(0\right)}(1),x_{1}^{\left(0\right)}(2),\ldots ,x_{1}^{\left(0\right)}(k)\right\};r=1,2,3,\ldots ,k$$

All features or dimensions used as independent variables of the system are formed into a sequence according to Equation (8).

(8) $$x_{i}^{\left(0\right)}\left(r\right)=\left\{x_{i}^{\left(0\right)}(1),x_{i}^{\left(0\right)}(2),\ldots ,x_{i}^{\left(0\right)}(r)\right\};i=1,2,3,\ldots ,N\;\mathrm{and}\;r=1,2,3,\ldots ,k$$

Step 2: To eliminate the uncertainties in the original data, we generate $x_{i}^{\left(0\right)}\left(r\right)$ using the accumulating generation operation (AGO).

The first-order accumulation generation operation (1-AGO) is performed from $x_{i}^{\left(0\right)};i=1,2,3,\ldots ,N$ as in Equation (9).

(9) $$x_{i}^{\left(1\right)}(r)=\sum_{j=1}^{k}x_{i}^{\left(0\right)}(j);k=1,2,3,\ldots ,r;i=1,2,3,\ldots ,N$$

Step 3: Evaluate the background value of $z_{i}^{\left(1\right)}(r)$ constructed by the generation method based on the average rate of two adjacent $x_{i}^{\left(1\right)}(r)$ datasets in Equation (10).

(10) $$z_{i}^{\left(1\right)}(r)=0.5\left(x_{i}^{\left(1\right)}(r-1)+x_{i}^{\left(1\right)}\left(r\right)\right),r=1,2,3,\ldots ,k$$

Step 4: Building a grey equation generation model in Equation (11).

(11) $$x_{i}^{\left(1\right)}\left(r\right)=\left\{x_{i}^{\left(1\right)}(1),x_{i}^{\left(1\right)}(2),\ldots ,x_{i}^{\left(1\right)}(k)\right\};r=1,2,3,\ldots ,k;i=1,2,3,\ldots ,N$$

Furthermore, for each pair of values, $x_{1}^{\left(0\right)}\left(r\right)$, $z_{i}^{\left(1\right)}(r)$ and $x_{i}^{\left(1\right)}(r)$ are formed to apply the grey differential equation in GM (1, N). However, before forming GM (1, N) the meaning of the grey differential equation GM (1, N) must be known as in Equation (12).

(12) $$\frac{dx_{1}^{\left(1\right)}(t)}{dt}+b_{1}x_{1}^{\left(1\right)}(t)=b_{2}x_{2}^{\left(1\right)}(t)+b_{3}x_{3}^{\left(1\right)}(t)+\ldots +b_{N}x_{N}^{\left(1\right)}(t)$$

where coefficient b_1_ is the coefficient of grey development, and b_2_, b_3_, …, b_N_ are the respective coefficients corresponding to the corresponding series. The coefficients b_1_, b_2_, …, b_N_ are model parameters to be estimated.

The grey derivative for the first-order grey differential equation with 1-AGO is conventionally represented as in Equations (13) and (14).

(13) $$\frac{dx_{1}^{\left(1\right)}(t)}{dt}\underset{\Delta t\to 0}{=\lim}\frac{x_{1}^{\left(1\right)}(t+\Delta t)-x_{1}^{\left(1\right)}(t)}{\Delta t}$$

(14) $$\frac{dx_{1}^{\left(1\right)}(t)}{dt}=\frac{\Delta x_{1}^{\left(1\right)}(t)}{\Delta t}=x_{1}^{\left(1\right)}(t+1)-x_{1}^{\left(1\right)}(t)=x_{1}^{\left(0\right)}(t+1)$$

when Δt → 1.

Step 5: Background values from $\frac{dx_{1}^{\left(1\right)}(t)}{dt}$, $x_{1}^{\left(1\right)}\left(t\right)$ are taken as the mean of $x_{1}^{\left(1\right)}\left(t\right)$ and $x_{1}^{\left(1\right)}(t+1)$ respectively, while $x_{j}^{\left(1\right)}\left(t\right)$, j = 2, 3, …, n is taken as $x_{j}^{\left(1\right)}\left(t\right)$, j = 2, 3, …, n.

The least squares solution for the model parameters of GM (1, N) in Equation (12) where t from 1 to r is in Equation (15).

(15) $${\left[\begin{array}{cccc} b_{1} & b_{2} & \ldots & b_{N} \end{array}\right]}^{T}={\left(B^{T}B\right)}^{-1}B^{T}Y_{N}$$

where

(16) $$B=\left[\begin{array}{ccccc} -\frac{1}{2}\left(x_{1}^{\left(1\right)}(1)-x_{1}^{\left(1\right)}(2)\right) & x_{2}^{\left(1\right)}(2) & x_{3}^{\left(1\right)}(2) & \ldots & x_{N}^{\left(1\right)}(2)\\ -\frac{1}{2}\left(x_{1}^{\left(1\right)}(2)-x_{1}^{\left(1\right)}(3)\right) & x_{2}^{\left(1\right)}(3) & x_{3}^{\left(1\right)}(3) & \ldots & x_{N}^{\left(1\right)}(3)\\ \vdots & \vdots & \vdots & \ldots & \vdots \\ -\frac{1}{2}\left(x_{1}^{\left(r-1\right)}(1)-x_{1}^{\left(1\right)}(r)\right) & x_{2}^{\left(1\right)}(r) & x_{3}^{\left(1\right)}(r) & \ldots & x_{N}^{\left(1\right)}(r) \end{array}\right]$$

and $Y_{N}(r)={\left[\begin{array}{cccc} x^{\left(0\right)}\left(2\right) & x^{\left(0\right)}\left(3\right) & \ldots & x^{\left(0\right)}\left(k\right) \end{array}\right]}^{T}.$ Then, the modeling value of the series prediction in Equation (16) is obtained as in Equation (17).

(17) $${\hat{x}}_{1}^{\left(1\right)}=\left[x_{1}^{\left(0\right)}\left(1\right)-\frac{1}{b_{1}}\sum_{i=1}^{n}b_{i}x_{i}^{\left(1\right)}\left(t\right)\right]e^{-b_{1}\left(t-1\right)}+\frac{1}{b_{1}}\sum_{i=1}^{n}b_{i}x_{i}^{\left(1\right)}\left(t\right)$$

From Equation (17), and by the inverse first-order accumulation generation operation (1-IAGO) of ${\hat{x}}_{1}^{\left(1\right)}$, the modeling value of ${\hat{x}}_{1}^{\left(0\right)}$ can be reduced in Equations (18) and (19).

(18) $${\hat{x}}_{1}^{\left(0\right)}\left(1\right)={\hat{x}}_{1}^{\left(1\right)}\left(1\right)=x_{1}^{\left(0\right)}\left(1\right)$$

(19) $${\hat{x}}_{1}^{\left(0\right)}\left(t\right)={\hat{x}}_{1}^{\left(1\right)}\left(t\right)-{\hat{x}}_{1}^{\left(1\right)}\left(t-1\right);t=2,3,\ldots$$

where t is a time (s), ${\hat{x}}_{1}^{\left(0\right)}$ is a prediction value, ${\hat{x}}_{1}^{\left(1\right)}$ is a first-order accumulation generation operation (1-IAGO).

## 4. Results and Discussion

### 4.1. Effect of Electrode Distance on Welding Distortion

**Figure 4:**
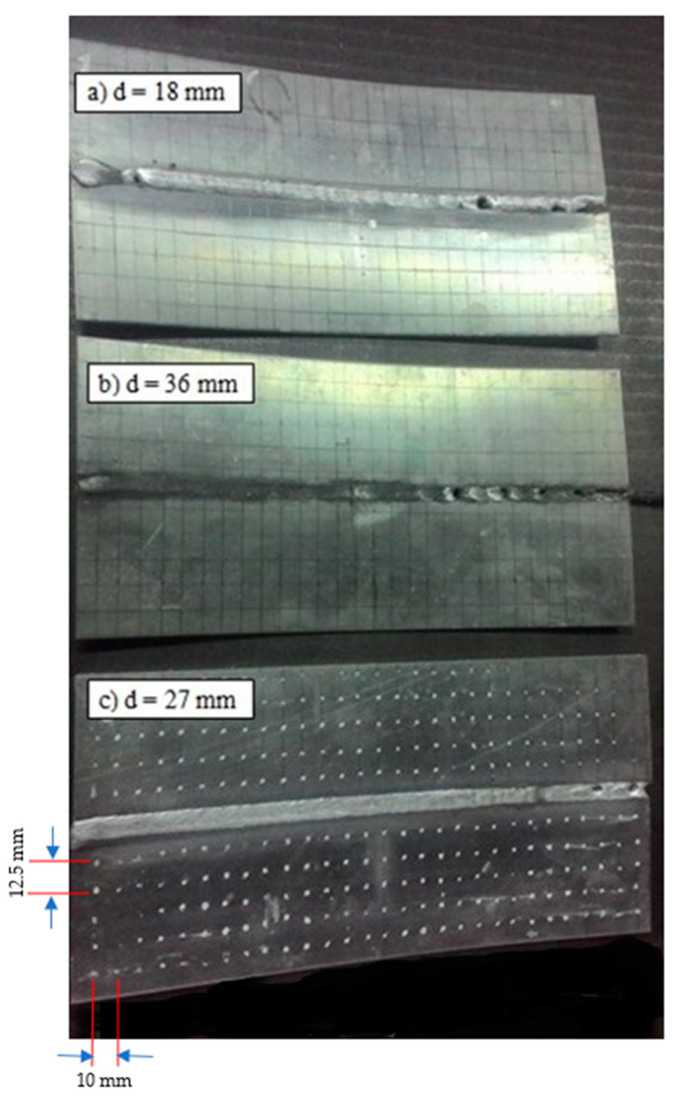
Specimen distortion in the welded plates.

Measurement of the distortion of the AA5052 aluminum plate aims to determine the curvature of the plate after experiencing the tandem MIG welding process caused by the uneven temperature distribution that occurs during the welding process. In the welding process, the weld metal will experience thermal problems. The results of out-of-plane distortion measurements for all welded plates versus electrode distance are depicted in Figure 4. During the welding process, the temperature of the weld metal will rise, but the amount of temperature increase varies from the highest temperature, namely the melting point of the metal, which occurs at the center of the weld, to the lowest temperature, which occurs at the edge of the metal, which is influenced by the ambient temperature. The temperature distribution, especially the peak temperature that occurs for each part of the weld metal and the time it takes to reach this temperature, greatly affects the properties of the weld metal. The properties of the weld metal that are affected by the temperature distribution include the shape of the macrostructure and the mechanical strength of the weld metal, such as hardness. In this study, the thermal cycle on the AA5052 aluminum plate occurs as a result of the magnitude of the temperature distribution in the weld metal, which is affected by the distance between the electrode and the center of the weld per unit time during the welding process.

The results of this study indicate that, at an electrode distance of 27 mm, torch interaction in tandem MIG welding will form a different thermal cycle than multi-run, which has two peaks, because the torch distance is very far. This phenomenon indicates that the interaction between torches in tandem MIG welding is very influential on the heat input that occurs; the closer the distance between the torches, the greater the heat input that occurs, so that the welding temperature becomes very high and the distortion will also be greater.

Based on Figure 4, showing the effect of the distance between the peak temperature of tandem MIG welding and the center of the weld, it is known that the closer the distance of the metal part of the weld to the center of the weld, the greater the temperature achieved, so that the part that reaches a higher peak temperature will experience a faster cooling process when the part that is reaching a lower peak temperature is still undergoing a heating process. The greater the peak temperature reached, the greater the cooling rate.

### 4.2. Vickers Hardness

The smallest distortion at an electrode distance of 27 mm was 1.4 mm. At an electrode distance of 27 mm, the plate may reach a proper peak temperature where the amount of heat input and dissipation rate are to those of electrode distances of 18 mm and 36 mm, resulting in greater distortion. Thus, this distance may be recommended for MIG tandem welding of a 5 mm thick plate of AA5052 material using ER5356 electrodes. Ghosh et al. [54] reported that the welding distortion of the V grove is commonly larger than that of an I grove. Therefore, in order to minimize welding distortion, it can be done by employing double-sided arc welding.

The Vickers hardness number (VHN) values of AA5052 tandem MIG welding with electrode distances of 18, 27, and 36 mm at the welding speed of 7 mm/s are shown in Table 2. The average VHN values in the base metal (BM), heat-affected zone (HAZ), and weld metal (WM) regions are presented. The highest relative VHN of 57 was found in the BM, while the lowest, 46, was found in the WM, showing good agreement with their respective grain sizes [51]. Finer grain size results in higher strength or hardness, with strength being proportional to $d^{-1/2}$, but according to Doksanovic et al. [55], their difference is not significant. Thus, this welding quality can be considered good.

The weld area is divided into three main parts, namely the weld metal, the heat-affected zone (HAZ), and the base metal, which is not affected by welding. The metal being welded and the HAZ zone will go through a number of temperature cycles during the welding operation. The thermal cycle will have an effect on the microstructure of the weld metal and HAZ, as the weld metal will go through a series of phase changes during the cooling process.

### 4.3. Thermal Cycle

**Figure 5:**
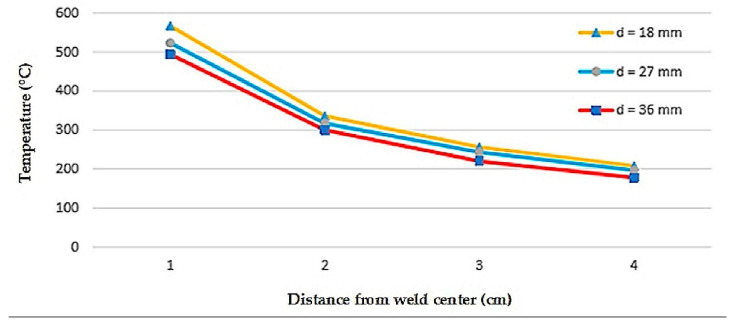
Peak temperature of tandem MIG welding.

Detailed results of the thermal cycle measurements during the welding processes for a welding speed of 7 mm/s are shown in Figure 5, while the peak temperatures TC1, TC2, TC3, and TC4 were presented in Table 3 for the three electrode distances of 18, 27, and 36 mm. The closer the electrode is to the weld center, the higher the peak temperature because heat input is concentrated in a smaller area, resulting in a longer heat dissipation time. The welding process produces a complicated thermal cycle in the weld. This thermal cycle causes changes in the micromaterial structure in the area around the weld (HAZ) and transient thermal stress, which eventually creates residual stress and distortion. Based on Table 3, it is found that the greater the thermal cycle, the greater the thermal stress. However, the resisting stresses tend to be the same for the same material parameters and dimensions. As a result, an increase in the thermal cycle will be accompanied by an increase in thermal stress, resulting in greater arc distortion.

Welding distortion is caused by shrinkage during the cooling process of the AA5052 aluminum plate material, which previously experienced expansion during the welding process. The factors that influence the above distortion include the hardness value and thermal temperature of the connection configuration and the MIG welding method.

The material is subjected to a temperature gradient, and, as long as thermal stress is produced, it tends to expand differently. In the aforementioned procedure, the thermal cycle (TC3) and the effects of distortion happen 30 mm away from the weld’s center. This is due to the influence of the optimal welding electrode spacing in this study, which is 27 mm. Within that distance, the welding electrode liquid will flow and settle properly, so that the effect of the weld results will produce welds and translucency on the AA5052 aluminum material. In addition, it also produces a fine sprinkling of slag during the welding process.

According to Hernández et al. [56], the Rosenthal solution to the temperature distribution of a moving point heating element for thin plates in Equation (20) can be used to approximate the four types of weld thermal cycles depicted in Figure 5.

(20) $$T-T_{0}=\frac{q_{w}}{h{\left(4\pi k\rho ct\right)}^{1/2}}\exp\left(-\frac{r^{2}}{4\alpha t}\right)$$

where $q_{w}$ is heat input determined by calculating (*Q/v*), *Q* is heat energy, *v* is welding speed, $T_{0}$ is the temperature at the beginning of welding, ρc is the specific heat per unit volume, *k* is thermal conduction, α is thermal diffusivity which is equal to *k*/ρc, *h* is the thickness of plate, *r* is the radial or lateral distance from the weld, and *t* is time.

The amount of heat input is relatively low, resulting in a low peak temperature, whereas the heat input of tandem welding is close to the total amount of heat input generated by the torches. In a heat sink condition, the cooling system dissipates a significant amount of heat input, resulting in lower peak temperatures.

### 4.4. Integrated GRA and GM (1, 6)

The grey analysis confirms and finds that the variable parameters that influence distortion from experimental studies are Vickers hardness values and thermal cycles. Both are considered the maximum and average reactions. GRA first normalizes the experimental results and then calculates the grey relational coefficients from the normalized data to reflect the relationship between the desired and actual experimental data, as illustrated in Table 4. GRG can be effectively adapted to solve complex interrelationships among defined performance characteristics.

This is because, at that distance, a process called thermal cycling happens. This involves heating and cooling a material until it goes through molecular reorganization, which tightens or optimizes the particle structure of the whole material, removes stress, and makes it denser and more uniform so that flaws or imperfections are minimized. This is why the electrode distance of 27 mm at TC3 gives the AA5052 aluminum plate a more even temperature distribution.

The grey prediction model GM (1, 6) is used to anticipate distortion analysis in tandem MIG welding and has a high prediction accuracy [57]. In this research, the modeling values in GM (1, 6) are theoretically the precise solution. There must be a strong correlation between the expected series and the associated series, and the indicator must be highly indicative of the predicted series in order to achieve high prediction accuracy for a dynamic system. Instead of the existing grey relational grade analysis, the upgraded grey relational grade analysis is employed by GM (1, 6) to examine the suitable entries of the projected series and the associated series used for model development.

The following can be made into a row using the results of modeling the welding results at an electrode distance of 27 mm and the distortion value that was determined from the following data:

$$x_{1}^{\left(0\right)}\left(r\right)=\left\{0,0.14,0.33,0.38,0.51,0.63,0.75,0.87,0.99,1.09,1.24,1.27,1.35,1.43,1.5,1.55,1.6,1.64,1.67,1.69\right\}$$

Then, here are the results of putting together the list of features or the list of independent variables:

$$\begin{array}{l} x_{2}^{\left(0\right)}\left(r\right)=\left\{48,50,49,48,48.5,53,52,54,55,55,59,60,59,59,59,62,65,66,60,60\right\}\\ x_{3}^{\left(0\right)}\left(r\right)=\left\{\begin{array}{l} 29.4776,29.4566,29.4788,29.4823,29.4814,29.4678,29.4987,29.4869,29.4853,29.4779,\\ 29.4787,29.4722,29.4697,29.4704,29.4687,29.4772,29.486,29.4807,29.4669,29.4818 \end{array}\right\}\\ x_{4}^{\left(0\right)}\left(r\right)=\left\{\begin{array}{l} 29.1908,29.1754,29.1899,29.1936,29.1791,29.1904,29.1958,29.1903,29.1961,29.1888,\\ 29.1954,29.1835,29.1886,29.1888,29.1932,29.1844,29.1987,29.1987,29.1919,29.1849 \end{array}\right\}\\ x_{5}^{\left(0\right)}\left(r\right)=\left\{\begin{array}{l} 29.3362,29.3087,29.3201,29.3098,29.3175,29.3228,29.2989,29.33,29.3223,29.3048,\\ 29.3188,29.3259,29.3239,29.3305,29.3292,29.3212,29.3167,29.3223,29.3076,29.3287 \end{array}\right\}\\ x_{6}^{\left(0\right)}\left(r\right)=\left\{\begin{array}{l} 29.2944,29.2655,29.254,29.2736,29.2958,29.2666,29.2844,29.2778,29.2826,29.2688,\\ 29.2613,29.2738,29.2764,29.2732,29.2843,29.2852,29.2743,29.2798,29.2753.29.2712 \end{array}\right\} \end{array}$$

The result of the first-order (1-AGO) accumulation generation operation for each variable is

$$X_{1}^{\left(1\right)}=\left[0\;0.14;0.47;0.85;1.36;1.99;2.74;3.61;4.6;5.69;6.93;8.2;9.55;10.98;12.48;14.03;15.63;17.27;18.94;20.63\right]$$

and

$$X_{i}^{\left(1\right)}=\left[\begin{array}{cccccccccccccccccccc} 48 & 98 & 147 & 195 & 243.5 & 296.5 & 348.5 & 402.5 & 457.5 & 512.5 & 571.5 & 631.5 & 690.5 & 749.5 & 808.5 & 870.5 & 935.5 & 1001.5 & 1061.5 & 1121.5\\ 29.4776 & 58.9342 & 88.413 & 117.8953 & 147.3767 & 176.8446 & 206.3432 & 235.8301 & 265.3154 & 294.7933 & 324.272 & 353.7442 & 383.2139 & 412.6842 & 442.153 & 471.6302 & 501.1162 & 530.5968 & 560.0637 & 589.5455\\ 29.1908 & 58.3662 & 87.5561 & 116.7496 & 145.9287 & 175.1191 & 204.3149 & 233.5051 & 262.7013 & 291.8901 & 321.0855 & 350.2689 & 379.4575 & 408.6463 & 437.8395 & 467.0239 & 496.2226 & 525.4213 & 554.6132 & 583.7981\\ 29.3362 & 58.6449 & 87.9649 & 117.2747 & 146.5922 & 175.915 & 205.2139 & 234.5439 & 263.8661 & 293.171 & 322.4897 & 351.8157 & 381.1396 & 410.4701 & 439.7993 & 469.1205 & 498.4372 & 527.7595 & 557.067 & 586.3957\\ 29.2944 & 58.5599 & 87.8138 & 117.0874 & 146.3833 & 175.6499 & 204.9343 & 234.2122 & 263.4948 & 292.7636 & 322.0249 & 351.2987 & 380.575 & 409.8483 & 439.1325 & 468.4177 & 497.692 & 526.9719 & 556.2472 & 585.5184 \end{array}\right]$$

where *i* = 2, 3, …, 20.

Furthermore, the mean sequence of $x_{1}^{\left(1\right)}(t)$ and $x_{1}^{\left(1\right)}(t+1)$ can be seen in the following matrix.

$$Z_{1}^{\left(1\right)}=\left[0.07;0.305;0.66;1.105;1.675;2.365;3.175;4.105;5.145;6.31\;;7.565;8.875;10.265;11.73;13.255;14.83;16.45;18.105;19.785\right]$$

By using matrix *B* in Equation (16), we get B:

$$B=\left[\begin{array}{cccccc} -0.07 & 98 & 58.9342 & 58.3662 & 58.6449 & 58.5599\\ -0.305 & 147 & 88.413 & 87.5561 & 87.9649 & 87.8138\\ -0.66 & 195 & 117.8953 & 116.7496 & 117.2747 & 117.0874\\ -1.105 & 243.5 & 147.3767 & 145.9287 & 146.5922 & 146.3833\\ -1.675 & 296.5 & 176.8446 & 175.1191 & 175.915 & 175.6499\\ -2.365 & 348.5 & 206.3432 & 204.3149 & 205.2139 & 204.9343\\ -3.175 & 402.5 & 235.8301 & 233.5051 & 234.5439 & 234.2122\\ -4.105 & 457.5 & 265.3154 & 262.7013 & 263.8661 & 263.4948\\ -5.145 & 512.5 & 294.7933 & 291.8901 & 293.171 & 292.7636\\ -6.31 & 571.5 & 324.272 & 321.0855 & 322.4897 & 322.0249\\ -7.565 & 631.5 & 353.7442 & 350.2689 & 351.8157 & 351.2987\\ -8.875 & 690.5 & 383.2139 & 379.4575 & 381.1396 & 380.575\\ -10.265 & 749.5 & 412.6842 & 408.6463 & 410.4701 & 409.8483\\ -11.73 & 808.5 & 442.153 & 437.8395 & 439.7993 & 439.1325\\ -13.255 & 870.5 & 471.6302 & 467.0239 & 469.1205 & 468.4177\\ -14.83 & 935.5 & 501.1162 & 496.2226 & 498.4372 & 497.692\\ -16.45 & 1001.5 & 530.5968 & 525.4213 & 527.7595 & 526.9719\\ -18.105 & 1061.5 & 560.0637 & 554.6132 & 557.067 & 556.2472\\ -19.785 & 1121.5 & 589.5455 & 583.7981 & 586.3957 & 585.5184 \end{array}\right]$$

Then the coefficient value of the independent variables in Equation (15) can be solved and the grey equation generation model is obtained, as shown in Equation (21).

(21) $$\frac{dx_{1}^{\left(1\right)}(t)}{dt}-0.0462x_{1}^{\left(1\right)}(t)=-0.0077x_{2}^{\left(1\right)}(t)+3.6606x_{3}^{\left(1\right)}(t)-1.8749x_{4}^{\left(1\right)}(t)+1.1569x_{5}^{\left(1\right)}(t)-2.9587x_{6}^{\left(1\right)}(t)$$

where $b_{1}=-0.0462;b_{2}=-0.0077;b_{3}=3.6606;b_{4}=-1.8749;b_{5}=1.1569;b_{6}=-2.9587$.

With the least squares for the model parameters of GM (1, 6) in Equation (21) using Equations (15) and (16). Furthermore, with $Y_{5}(r)={\left[\begin{array}{cccc} 2.8539 & 3.4928 & 3.7879 & 4.1057 \end{array}\right]}^{T}$ then, the modeling value of the series prediction is obtained as in Equation (22).

(22) $${\hat{x}}_{1}^{\left(1\right)}=\left[x_{1}^{\left(0\right)}\left(1\right)+\frac{1}{0.0462}\sum_{i=1}^{n}b_{i}x_{i}^{\left(1\right)}\left(t\right)\right]e^{0.0462\left(t-1\right)}-\frac{1}{0.0462}\sum_{i=1}^{n}b_{i}x_{i}^{\left(1\right)}\left(t\right),\;\mathrm{where}\;i=2,3,4,\ldots ,12$$

where

$$\sum_{i=2}^{6}b_{i}x_{i}^{\left(1\right)}\left(t\right)=-0.0077x_{2}^{\left(1\right)}(t)+3.6606x_{3}^{\left(1\right)}(t)-1.8749x_{4}^{\left(1\right)}(t)+1.1569x_{5}^{\left(1\right)}(t)-2.9587x_{6}^{\left(1\right)}(t)$$

**Figure 6:**
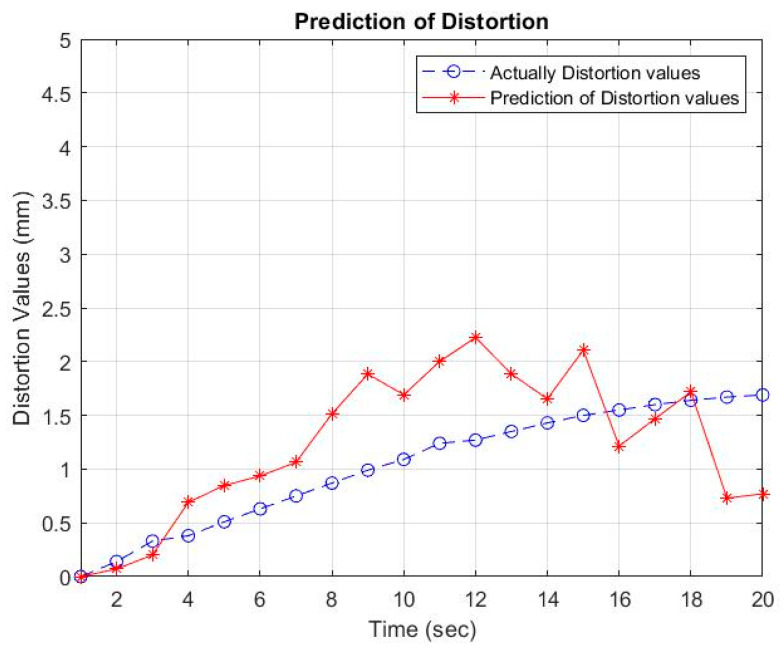
Comparison of predictions and experiments of distortion values at an electrode distance of 18 mm.

**Figure 7:**
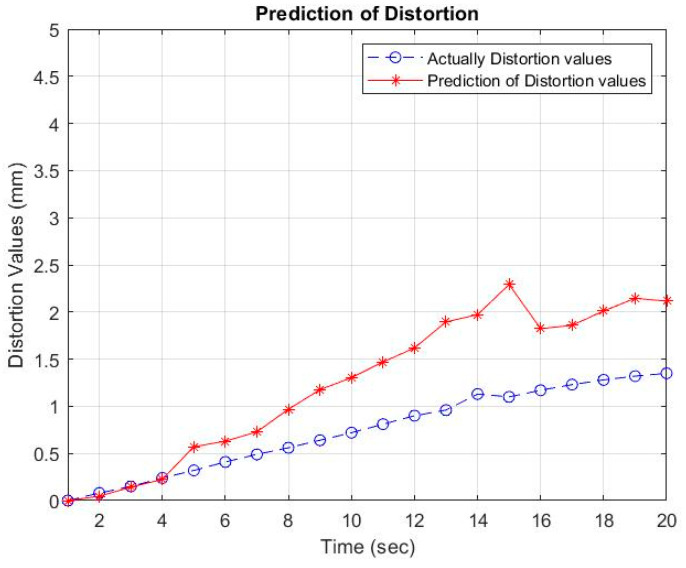
Comparison of predictions and experiments of distortion values at an electrode distance of 27 mm.

**Figure 8:**
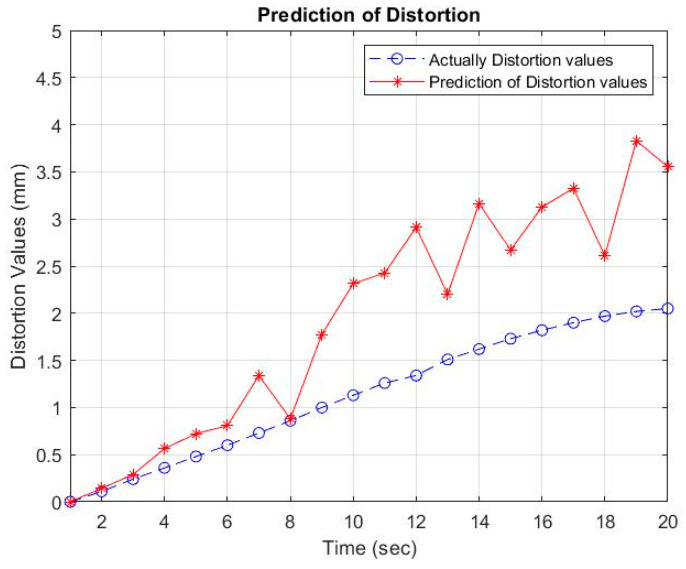
Comparison of predictions and experiments of distortion values at an electrode distance of 36 mm.

Then, using the inverse-generating operation of the first-order accumulation (1-IAGO) of ${\hat{x}}_{1}^{\left(1\right)}$, the modeling value of ${\hat{x}}_{1}^{\left(0\right)}$ can be obtained, as depicted in Table 5 and Figure 6, Figure 7 and Figure 8.

The GM (1, 6) values are close to the empirical values. This is because the pattern of empirical data follows an upward trend and does not experience fluctuations, so the two models can be used as a reference for looking for random data analysis. The overlapping distortion between the actual data and GM (1, 6) is shown in Figure 7 with a welding electrode distance of 27 mm.

Based on the prediction results using GM (1, 6), a comparison can be made between the actual data and the predicted results using GM (1, 6). A comparison graph of the data pattern with the welding electrode distance variable is shown in Figure 6, Figure 7 and Figure 8. The distortion value in the actual results is influenced by several factors, including the Vickers hardness and the thermal cycle temperature at a distance of 30 mm from the welding point. Mechanical properties, which consist of Vickers hardness and thermal cycles as independent variables that are controlled by electrode distance as the controlling variable, affect the results of distortion on the AA5052 aluminum plate. The arrangement of the welding electrode distance is able to minimize welding distortion with thin plates.

### 4.5. Evaluation Process

The results applying GM (1, 6) on tandem MIG welding indicate the model’s effectiveness and prediction accuracy. However, an evaluation is required to measure the error rates between the predicted and the actual values. This is shown in Table 6. The evaluation processes are as follows:

Step 1: Root mean square error (RMSE)

In this research, the root mean square error (RMSE) approach was employed to evaluate the performance of the model GM (1, 6). RMSE is sometimes known as the root mean square deviation (RMSD); its mathematical expression is similar to SD in that RMSE pertains to n data points rather than n^−1^. The value generated by the RMSE is the average squared value of the number of errors in the prediction model. RMSE is a technique that is easy to implement and has been widely used in various studies related to prediction or forecasting [58,59]. The mathematical equation RMSE is shown in Equation (23).

(23) $$RMSE=\sqrt{\frac{1}{n}\sum_{i=1}^{n}{\left(x_{1}^{0}-{\hat{x}}_{1}^{\left(0\right)}\right)}^{2}}$$

Step 2: Mean square error (MSE)

Calculating the MSE value is similar to calculating the RMSE. MSE is an error calculation method that is calculated by adding up the squared errors and then dividing it by the number of data or periods used [60]. At this stage, the greater the error value, the greater the resulting MSE value shown in Equation (24).

(24) $$MSE=\frac{1}{n}\sum_{i=1}^{n}{\left(x_{1}^{0}-{\hat{x}}_{1}^{\left(0\right)}\right)}^{2}$$

Step 3: Mean absolute percentage error (MAPE)

The mean absolute percentage error (MAPE) is the average value of the absolute difference between the predicted and actual values, expressed as a percentage of the realized value. The use of the mean absolute percentage error (MAPE) in forecasting results can demonstrate the amount of accuracy of forecasting and realization data. Equation (25) can be used to calculate MAPE value [61].

(25) $$MAPE=\frac{1}{n}\sum_{i=1}^{n}\left(\left|\frac{x_{1}^{0}-{\hat{x}}_{1}^{\left(0\right)}}{x_{1}^{0}}\right|\times 100\%\right)$$

where

$x_{1}^{0}$ = actual value
${\hat{x}}_{1}^{\left(0\right)}$ = prediction value
*i* = order of data in the database
*n* = datasets

This criterion is similar to the RMSE in its measurement. Nevertheless, it is more reliable than MSE because it is less sensitive to extreme values. All distance measurements (RMSE and MSE) are equivalent and help to quantify the approximated solution’s accuracy in comparison to the simulated data between actual and prediction in welding distortion. Low values for these criteria indicate that the estimated model is reasonably close to the true value. The closer the RMSE values are to zero and 1, respectively, the more accurate the model results will be. MAPE provides information on how much the forecast error is compared to the actual value of the series. MAPE represents the average absolute percentage error of each entry in a data set to calculate how accurate the predicted value is compared to the true value. MAPE is a direct metric, with the results of the data above showing that with electrode distances of 18, 27, and 36 mm, the MAPE values are 0.52%, 1.21%, and 1.35%, respectively. It can be interpreted that the value represents the average deviation between the predicted value and the actual value, regardless of whether the deviation is positive or negative.

## 5. Conclusions

Based on the results of research and testing of tandem MIG welding with electrode distances of 18, 27, and 36 mm on AA5052 aluminum material with a welding speed of 7 mm/s, it can be concluded that the smallest distortion of the AA5052 aluminum plate occurs at a welding electrode distance of 27 mm. This is influenced by the Vickers hardness level in the area, with that of the base metal (BM) being 56.53 VHN, of the heat-affected zone (HAZ) being 52.75 VHN, and of the weld metal (WM) being 48.46 VHN. The level of hardness at that distance is also affected by the thermal cycle temperature at a distance of 30 mm from the weld metal (WM) because the heat input is more concentrated in the WM area. The effect of the distance between the welding electrodes in this research indicated that the heat input is directly proportional to the value of the shrinkage voltage such that, the higher the heat input, the higher the shrinkage voltage. This is consistent with the distortion of large tandem welding results caused by high heat input. By using GRA analysis and the GM (1, 6), the effect of welding distortion is found for an electrode distance of 27 mm with actual and predicted values that coincide with each other. Process evaluation results the predictions for welding distortions do not experience fluctuating numbers; thus, the GM (1, 6) model can be used as a predictive model for welding distortions of 5052 aluminum plates. The results of the evaluation were carried out using the MAPE method to determine the ability of the model used to see the difference in actual and predicted values. The MAPE value at an electrode distance of 18 mm is 0.52%, while at an electrode distance of 27 mm, it is 1.21%, and at 36 mm, it is 1.52%. The optimum value from the experimental results shows that the smallest distortion occurs at the welding electrode distance of 27 mm. However, GM (1, 6) cannot show the optimal value of welding distortion based on electrode distance. In the future, more research can be done on how to use the GM (1, N) model to find the best electrode distance in the welding process to achieve optimal distortion.

## Figures and Tables

**Table 1:** Different welding techniques.

No	Types of Welding	Advantages	Disadvantages
1	Arc welding	High strength weld High weld speed Low welding equipment	Low efficiency Difficult to weld thin materials
2	Gas welding	Low equipment cost Portable equipment Highly skilled welders are not required	Low weld quality Not suitable for thick sections
3	Resistance welding	Low processing cost Easy to implement Welding dissimilar materials are possible	Comparatively low welding strength Aesthetics are not good near the weld area
4	Solid-State welding	Weld dissimilar materials and thermoplastic is possible Weld joints are free from microstructure defects	Difficult to set up Surface preparation is required
5	Energy beam welding	Weld strength up to 95% is achievable A vacuum environment eliminates impurities	EBW requires vacuum conditions to prevent dissipation of the electron beam
6	Laser welding	High-quality welds Narrow and deep welds are possible Automation is easy to implement	High initial equipment and maintenance cost Sometimes cracking is a concern due to the high cooling rate

**Table 2:** Average Vickers Hardness Number (VHN) of tandem MIG welding.

Welding Speed (mm/s)	Electrode Distance (mm)	Average Vickers Hardness Number (VHN)		
		BM	WM	HAZ
7	18	56	49	53
	27	57	48	53
	36	55	46	52

**Table 3:** Peak temperature in the thermal cycle (TC).

Electrode Distance	Peak Temperature (°C) of Tandem MIG Welding at Welding Speed of 7 mm/s			
	TC1	TC2	TC3	TC4
18 27 36	567 523 494	336 317 300	256 243 220	208 197 178

**Table 4:** GRG for different electrode distances.

Experiment	Grey Relational Grade			Rank
	18 (mm)	27 (mm)	36 (mm)	
1	0.7030	0.7192	0.6998	2
2	0.5459	0.5863	0.6238	5
3	0.6972	0.6444	0.6138	4
4	0.7579	0.7809	0.6237	1
5	0.6640	0.7513	0.6228	3

(Experiment: 1 = Distortion-VHN; 2 = Distortion-TC1; 3 = Distortion-TC2; 4 = Distortion-TC3; 5 = Distortion-TC4).

**Table 5:** Welding distortion for different electrode distances.

Length (mm)	Distortion in Variable Electrode Distance					
	18 mm		27 mm		36 mm	
	$x_{1}^{0}$	${\hat{x}}_{1}^{\left(0\right)}$	$x_{1}^{0}$	${\hat{x}}_{1}^{\left(0\right)}$	$x_{1}^{0}$	${\hat{x}}_{1}^{\left(0\right)}$
0	0.00	0	0.00	0	0.00	0
10	0.14	0.07	0.08	0.05	0.11	0.14
20	0.33	0.20	0.15	0.14	0.24	0.29
30	0.38	0.69	0.24	0.23	0.36	0.56
40	0.51	0.85	0.32	0.57	0.48	0.72
50	0.63	0.94	0.41	0.63	0.60	0.81
60	0.75	1.06	0.49	0.73	0.73	1.34
70	0.87	1.51	0.56	0.97	0.86	0.88
80	0.99	1.88	0.64	1.18	1.00	1.78
90	1.09	1.69	0.72	1.30	1.13	2.31
100	1.24	2.00	0.81	1.47	1.26	2.43
110	1.27	2.22	0.90	1.62	1.34	2.91
120	1.35	1.89	0.96	1.90	1.51	2.20
130	1.43	1.65	1.13	1.97	1.62	3.16
140	1.50	2.11	1.10	2.29	1.73	2.67
150	1.55	1.21	1.17	1.82	1.82	3.13
160	1.60	1.47	1.23	1.86	1.90	3.33
170	1.64	1.72	1.28	2.01	1.97	2.61
180	1.67	0.73	1.32	2.15	2.02	3.83
190	1.69	0.77	1.35	2.12	2.05	3.55

**Table 6:** Root mean square error (RMSE) for actual and prediction data.

Variable Electrode Distance (mm)	$\sum_{i=1}^{n}{\left(x_{1}^{0}-{\hat{x}}_{1}^{\left(0\right)}\right)}^{2}$	MSE	RMSE	MAPE (%)
18	6.061065231	0.303053	0.550503	0.52
27	7.545394	0.37727	0.614223	1.21
36	19.77073	0.988537	0.994252	1.35

## Data Availability

The data used to support the finding of this study are included within the article.
